# Crosstalk between SOX2 and cytokine signaling in endometrial carcinoma

**DOI:** 10.1038/s41598-018-35592-0

**Published:** 2018-12-03

**Authors:** Chang-Jung Lee, Pi-Lin Sung, Ming-Han Kuo, Min-Hwa Tsai, Cheng-Kuang Wang, Shien-Tung Pan, Yi-Jen Chen, Peng-Hui Wang, Kuo-Chang Wen, Yu-Ting Chou

**Affiliations:** 1 Department of Life Science and Institute of Biotechnology, National Tsing-Hua University, HsinChu, 300 Taiwan (R.O.C.); 2 Department of Obstetrics and Gynecology, Taipei Veterans General Hospital, Taipei, 112 Taiwan (R.O.C.); 3 Department of Obstetrics and Gynecology, National Yang-Ming University, Taipei, 112 Taiwan (R.O.C.); 4 Department of Medical Technology, Jen-Teh Junior College of Medicine, Nursing and Management, Miaoli, 356 Taiwan (R.O.C.); 5 Department of Pathology, Tungs’ Taichung MetroHarbor Hospital, Taichung, 433 Taiwan (R.O.C.)

## Abstract

Endometrial carcinoma is a cancer derived from oncogenesis of the regenerating uterine cavity, in which cytokine stimulation shapes cell differentiation and tissue remodeling. Expression of the stem cell factors *SOX2*, *OCT4*, *NANOG*, and *MYC* has been linked to tumor malignancy in several cancers. However, how these stem cell factors crosstalk with cytokine signaling to promote malignancy in endometrial carcinoma is still elusive. Here we report that the expression of *SOX2* and *MYC*, but not that of *OCT4* and *NANOG*, correlate with poor histological differentiation and prognosis, while *SOX2* expression is negatively associated with *MYC* level. We found that *SOX2*-high endometrial carcinoma cells possessed a higher colony-forming ability than their *SOX2*-low counterparts, and knockdown of *SOX2* attenuated the colony-forming ability. We observed that SOX2 regulated EGFR expression in a SOX2–EGFR positive feedback loop. EGF stimulation induced *SOX2* expression and promoted migration of endometrial carcinoma cells, whereas TGF-β stimulation inhibited *SOX2* expression and attenuated the colony-forming ability. Immunohistochemistry analysis revealed that SOX2 expression correlated with lymph node infiltration of endometrial carcinoma. Our findings support that cytokine-induced stem cell factor *SOX2* possesses oncogenic properties, with the potential to serve as a prognostic biomarker in endometrial carcinoma.

## Introduction

Endometrial carcinoma, which arises from highly regenerating uterine cavity, is the most common gynecologic malignancy in developed countries^1^. Patients with endometrial carcinoma are often diagnosed with an early-stage disease, which indicates a good prognosis. Although endometrial carcinoma is a relatively manageable malignancy, this disease can range from easily controlled to aggressive. The patients diagnosed at a late-stage with endometrial tumors metastasizing to the lymph nodes or distant organs often have limited therapeutic options and experience poor survival outcomes^2^.

SOX2, OCT4, and NANOG are master transcription factors that form the regulatory circuitry to maintain stemness and prevent differentiation in embryonic stem cells (ESCs)^3^. These factors, once overexpressed with MYC, are able to reprogram differentiated somatic cells into pluripotent stem cells^4,5^. Moreover, it has been reported that poorly differentiated tumors exhibit highly activated ESC signaling^6^, while MYC expression reactivates the ESC program to cause tumor malignancy^7,8^. Accumulating evidence also indicates that the activation of endogenous interconnected auto-regulatory loops formed by OCT4, SOX2, and NANOG is important for tumor oncogenesis^9–11^.

SOX2 is expressed in several proliferative progenitor cells^12–14^. Lung progenitor cells, for example, express SOX2 to regulate tissue development and regeneration^14–16^. SOX2 is also detected in different types of tumors, including breast and lung tumors^17,18^. Moreover, *SOX2* amplification has been observed in lung squamous cell carcinoma^19^. While *SOX2* is reported to be hyper-methylated in endometrial carcinoma^20^, SOX2 expression is detected in this cancer^21,22^.

Distinct cytokines from microenvironments interact with stem cell signaling to shape cell differentiation, tissue development, and regeneration. The epidermal growth factor (EGF) activates the EGF receptor (EGFR) to promote SOX2 expression and thus induce self-renewal and proliferation in neuron precursor cells^13^. In the uterus, EGFR signaling is activated during the menstrual cycle to stimulate the proliferation of endometrium epithelial cells^23^. In contrast, TGF-β inhibits proliferation of uterine epithelial cells and mesenchymal stem cells, and loss of TGF-β receptors causes endometrial hyperplasia in a mouse model^24–26^. To date, how stem cell factors crosstalk with cytokine signaling to influence endometrial carcinoma malignancy remains unclear.

In this study, we observed that the expression of *SOX2* and *MYC*, but not that of *OCT4* and *NANOG*, in endometrial tumors is associated with poor histological differentiation and prognosis, suggesting the involvement of SOX2 in oncogenesis. Hence, we further characterized how SOX2 crosstalks with EGFR and TGF-β signaling to affect cancer cell growth and dissemination in endometrial carcinoma.

## Results

### *SOX2* expression correlates with poor histological grade and prognosis in endometrial carcinoma

**Figure 1:**
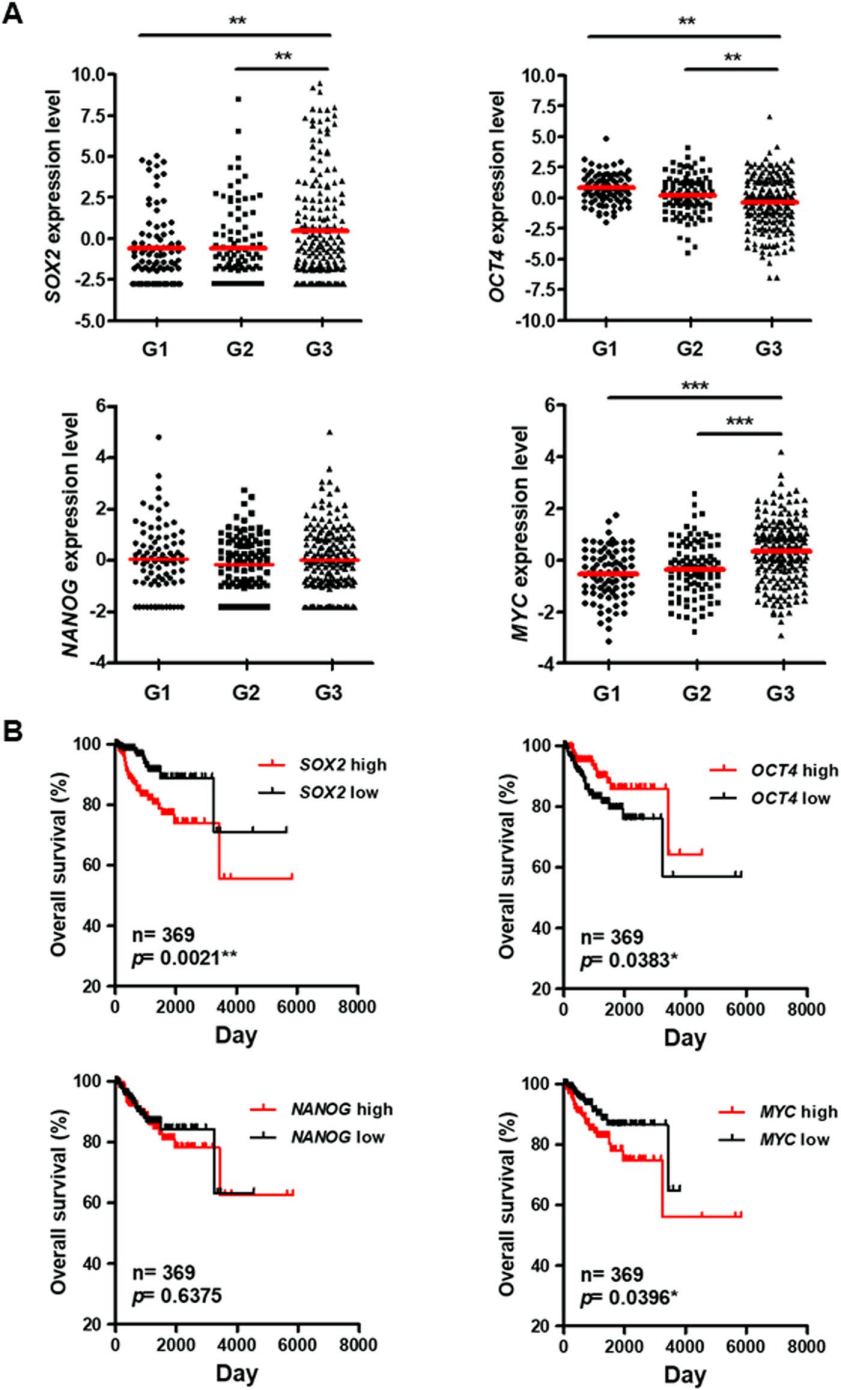
Correlation of *SOX2, OCT4*, *NANOG*, and *MYC* expression with histological grades and survival outcomes of endometrial carcinoma. (**A**) Gene expression analysis of *SOX2* (upper left), *OCT4* (upper right), *NANOG* (lower left), and *MYC* (lower right) expression with histological grades of endometria carcinoma from TCGA_UCEC cohort. The significance was examined by Tukey’s Multiple Comparison Test followed by one way ANOVA. **P < 0.01; ***P < 0.001. (**B**) A Kaplan–Meier analysis to assess the correlation of *SOX2* (upper left), *OCT4* (upper right), *NANOG* (lower left), and *MYC* (lower right) expression with the overall survival of patients with endometrial carcinoma from TCGA_UCEC cohort. The significance was examined by log-rank test.

Because ESC signaling has been linked to tumor malignancy in different cancers, we correlated the expression of the key transcription factor genes *SOX2*, *OCT4*, and *NANOG*, and of the oncogene *MYC* with histological grades in primary endometrial carcinoma based on the TCGA_UCEC cohort^27^. We observed that both *SOX2 and MYC* expression were associated with high grade tumor histology, while *OCT4* expression correlated with low grade histology (Fig. 1A and Supplementary Figure S1A–D). A correlation analysis revealed that the expression of *SOX2* was negatively correlated with that of *MYC* and *OCT4* (Supplementary Figure S1E, S1F). Moreover, we found that both *SOX2* and *MYC* exhibited profound gene amplifications in 7.1% (n = 17) of the samples, compared to *NANOG* (0.4%) and *OCT4* (2.1%) (Table 1). A correlation analysis showed that both *SOX2* and *MYC* amplifications were significantly associated with advanced grade in endometrial tumors (Table 2). These data indicate the potential involvement of *SOX2* in the oncogenesis of endometrial carcinoma.

**Table 1:** Gene copy-number variation analysis of four stem cell factors in endometrial carcinoma from TCGA_UCEC cohort (n = 242).

Gene	Amplification	Deletion	Mutation
*SOX2*	17 (7.1%)	1 (0.4%)	3 (1.2%)
*OCT4*	5 (2.1%)	0 (0%)	1 (0.4%)
*NANOG*	1 (0.4%)	0 (0%)	2 (0.8%)
*MYC*	17 (7.1%)	0 (0%)	8 (3.3%)

**Table 2:** Correlation of *SOX2* and *MYC* amplifications with histological grade in endometrial carcinoma from TCGA_UCEC cohort (n = 242).

Parameter	SOX2			MYC		
	Diploid	Amplification	*P*	Diploid	Amplification	*P*
**Grade**						
G1	74 (100%)	0 (0%)	<0.001***	73 (98.6%)	1 (1.4%)	<0.001***
G2	72 (98.6%)	1 (1.4%)		72 (98.6%)	1 (1.4%)	
G3	79 (83.2%)	16 (16.8%)		80 (84.2%)	15 (15.8%)	

P value was examined by Pearson’s chi-square test.

To examine the potential of *SOX2*, *OCT4*, *NANOG*, and *MYC* as prognostic markers in endometrial carcinoma, we correlated their expression with survival outcomes in patients. The Kaplan–Meier survival analysis showed that *SOX2* and *MYC* expression correlated with a poor overall survival in patients, while high *OCT4* expression was associated with a good overall survival (Fig. 1B). Although univariate analysis showed that individual expression of *SOX2*, *OCT4*, and *MYC* correlated with survival, multivariate analysis revealed that *SOX2* and *MYC*, but not *OCT4*, could function as independent prognostic factors in predicting survival of endometrial carcinoma (Supplementary Table S1). Loss of ESR1 expression has been associated with high grade endometrial carcinoma^28^. We found that *ESR1* expression predicted good histological differentiation and survival in endometrial tumors in patients (Supplementary Figure S2A, S2B). We observed that *SOX2* expression negatively correlated with *ESR1* level (Supplementary Figure S2C). Moreover, a *SOX2*-high/*ESR1*-low signature predicts a worse survival outcome in patients (Supplementary Figure S2B, right). These findings suggest an oncogenic role of SOX2 with the potential utility as a prognostic biomarker in endometrial carcinoma.

### SOX2 regulates proliferation and cell cycle progression in endometrial carcinoma

**Figure 2:**
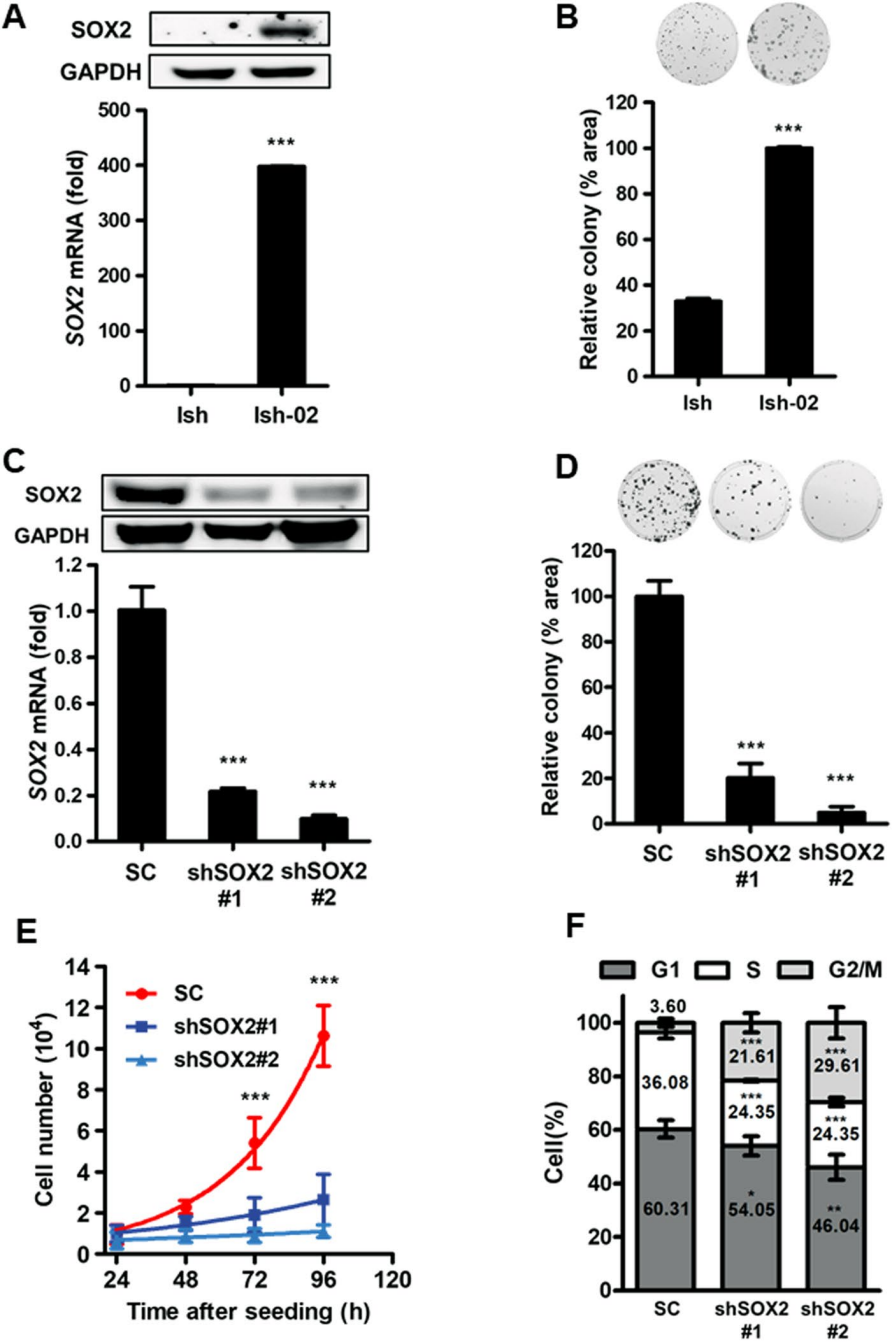
SOX2 contributes to cell proliferation and cell cycle progression in endometrial carcinoma. (**A**) Immunoblotting (upper) and qPCR (lower) analysis to measure SOX2 expression in Ishikawa (Ish, left) and Ishikawa-02 (Ish-02, right) endometrial carcinoma cells. ***P < 0.001. (**B**) A clonogenic analysis to compare the growth of *SOX2*-high Ishikawa-02 (Ish-02, right) to *SOX2*-low Ishikawa (Ish, left) cells. Colonies were subjected to crystal violet staining (upper) and quantified by ImageJ analysis (lower). ***P < 0.001. (**C**) Immunoblotting (upper) and qPCR (lower) analysis to measure the effect of SOX2 knockdown in Ishikawa-02 cells transduced with lentiviral particles encoding scrambled control (SC) or shSOX2. shSOX2#1 and shSOX2#2 target different regions of the *SOX2* mRNA. ***P < 0.001. (**D**) A clonogenic analysis to compare the growth of Ishikawa-02 cells transduced with lentiviral particles encoding scrambled control (SC) or shSOX2 for 14 days. Colonies were subjected to crystal violet staining (upper) and quantified by ImageJ analysis (lower). ***P < 0.001. (**E**) Trypan blue cell exclusion proliferation assay of Ishikawa-02 cells transduced with lentiviral particles encoding scrambled control (SC) or shSOX2 for 4 days. ***P < 0.001. (**F**) Flow cytometry cell cycle analysis with propidium iodide staining of Ishikawa-02 cells transduced with lentiviral particles encoding scrambled control (SC) or shSOX2. **P < 0.01; ***P < 0.001.

The above results suggest that *SOX2* predicts a poor prognosis in endometrial carcinoma and negatively correlates with *ESR1* expression. To identify *SOX2*-positive endometrial cancer cells for further oncogenic analysis, we checked SOX2 expression in Ishikawa (*ESR1*-positive) and its descendant Ishikawa-02 (*ESR1*-negative) cells. Immunoblotting and qPCR analysis showed that *SOX2* was highly expressed in Ishikawa-02 but not in Ishikawa cells. Moreover, clonogenic assays revealed that *SOX2*-high Ishikawa-02 cells exhibited a better colony-forming ability compared with Ishikawa cells (Fig. 2A,B). To study the role of SOX2 in cell proliferation, we knocked down *SOX2* in Ishikawa-02 by lentiviral transduction of shRNA targeting *SOX2* (Fig. 2C). Clonogenic assays and TrypanBlue cell exclusion analysis demonstrated that *SOX2*-silencing attenuated cell proliferation (Fig. 2D,E). Cell cycle analysis revealed that *SOX2* knockdown attenuated S phase and induced G2/M arrest (Fig. 2F). These data indicate that *SOX2* promotes cell proliferation and regulates cell cycle progression in endometrial carcinoma.

### A SOX2–EGFR positive feedback loop regulates proliferation of endometrial carcinoma cells

**Figure 3:**
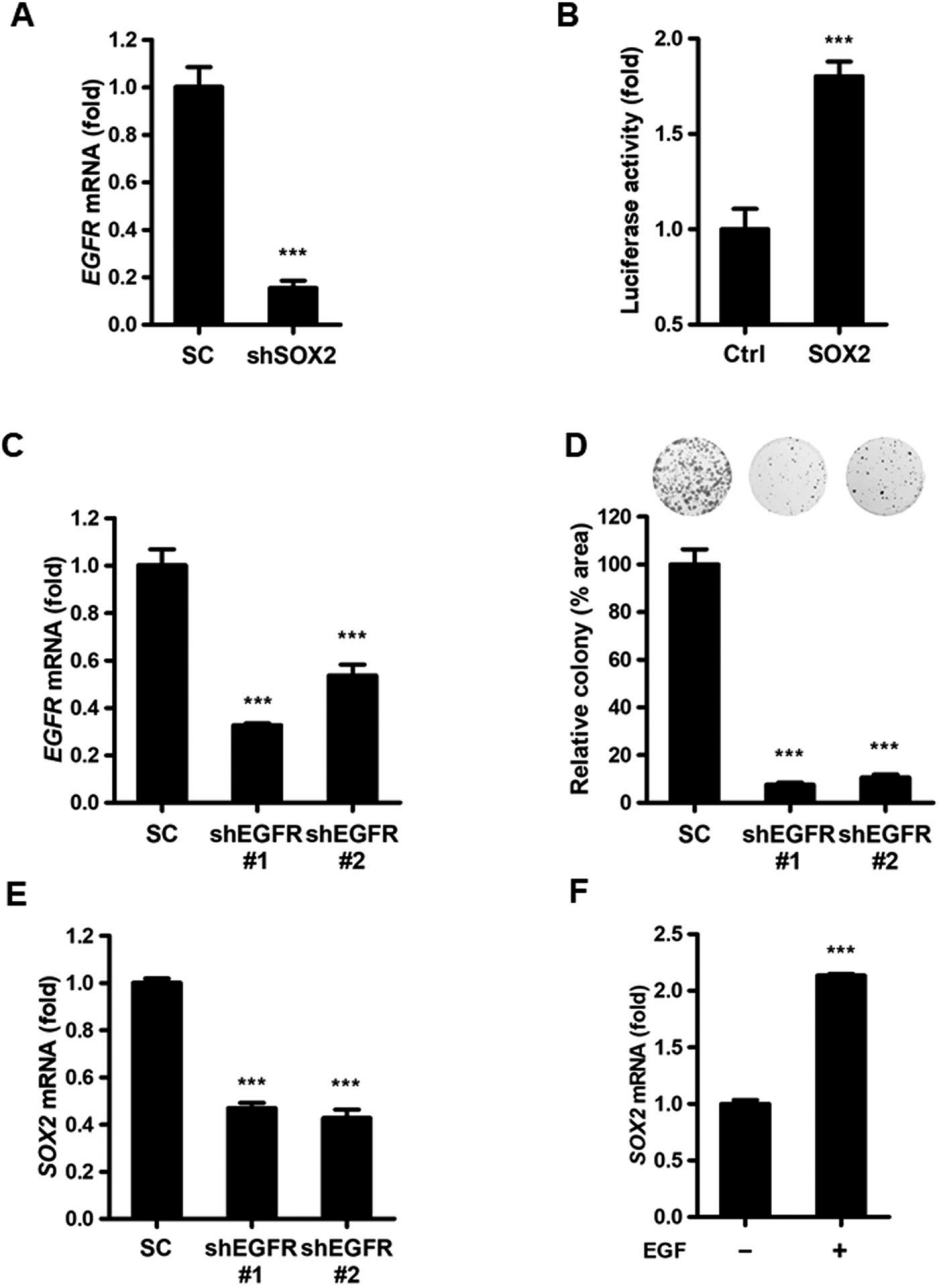
SOX2 regulates EGFR expression in a positive feedback loop. (**A**) qPCR analysis to measure *EGFR* expression in Ishikawa-02 cells transduced with lentiviral particles encoding scrambled control (SC) or shSOX2. ***P < 0.001. (**B**) A luciferase reporter assay to measure *EGFR* promoter reporter activity in HEK293T cells transfected with *EGFR*-pGL3 plus the vector encoding *SOX2* cDNA or empty control vector (Ctrl). Results were representative of three independent experiments and are expressed as the mean ± S.D. ***P < 0.001. (**C**) qPCR analysis to measure *EGFR* expression in Ishikawa-02 cells transduced with lentiviral particles encoding scrambled control (SC) or shEGFR. shEGFR#1 and shEGFR#2 target different regions of the *EGFR* mRNA. ***P < 0.001. (**D**) A clonogenic analysis to compare the growth of Ishikawa-02 cells transduced with lentiviral particles encoding scrambled control (SC) or shEGFRs for 14 days. Colonies were subjected to crystal violet staining (upper) and quantified by ImageJ analysis (lower). ***P < 0.001. (**E**) qPCR analysis to measure *SOX2* expression in Ishikawa-02 cells transduced with lentiviral particles encoding scrambled control (SC) or shEGFR. ***P < 0.001. (**F**) qPCR analysis to measure *SOX2* expression in Ishikawa-02 cells treated with or without EGF (10 ng/ml) for 24 hr. ***P < 0.001.

To identify genes involved in *SOX2*-mediated oncogenesis in endometrial carcinoma, a gene expression profiling assay was performed in *SOX2*-silenced Ishikawa-02 versus scrambled control cells, and the result was uploaded as GSE114582. qPCR analysis confirmed that *SOX2* knockdown attenuated *EGFR* expression (Fig. 3A). Luciferase reporter assays showed that *SOX2* expression enhanced *EGFR* promoter reporter activity (Fig. 3B). To further validate whether a SOX2–EGFR positive feedback loop is present in endometrial carcinoma, *EGFR* was knocked down in Ishikawa-02 (*SOX2*-high) cells (Fig. 3C). Clonogenic assays showed that *EGFR* silencing attenuated cell proliferation of Ishikawa-02 cells (Fig. 3D). A qPCR analysis showed that *EGFR* knockdown decreased *SOX2* expression levels, suggesting a positive feedback loop between SOX2 and EGFR (Fig. 3E). Moreover, EGF stimulation enhanced *SOX2* expression in Ishikawa-02 cells (Fig. 3F). These data indicate the participation of a SOX2–EGFR positive feedback loop in proliferation of endometrial carcinoma cells.

### TGF-β inhibits SOX2 expression and suppresses proliferation of endometrial carcinoma cells

**Figure 4:**
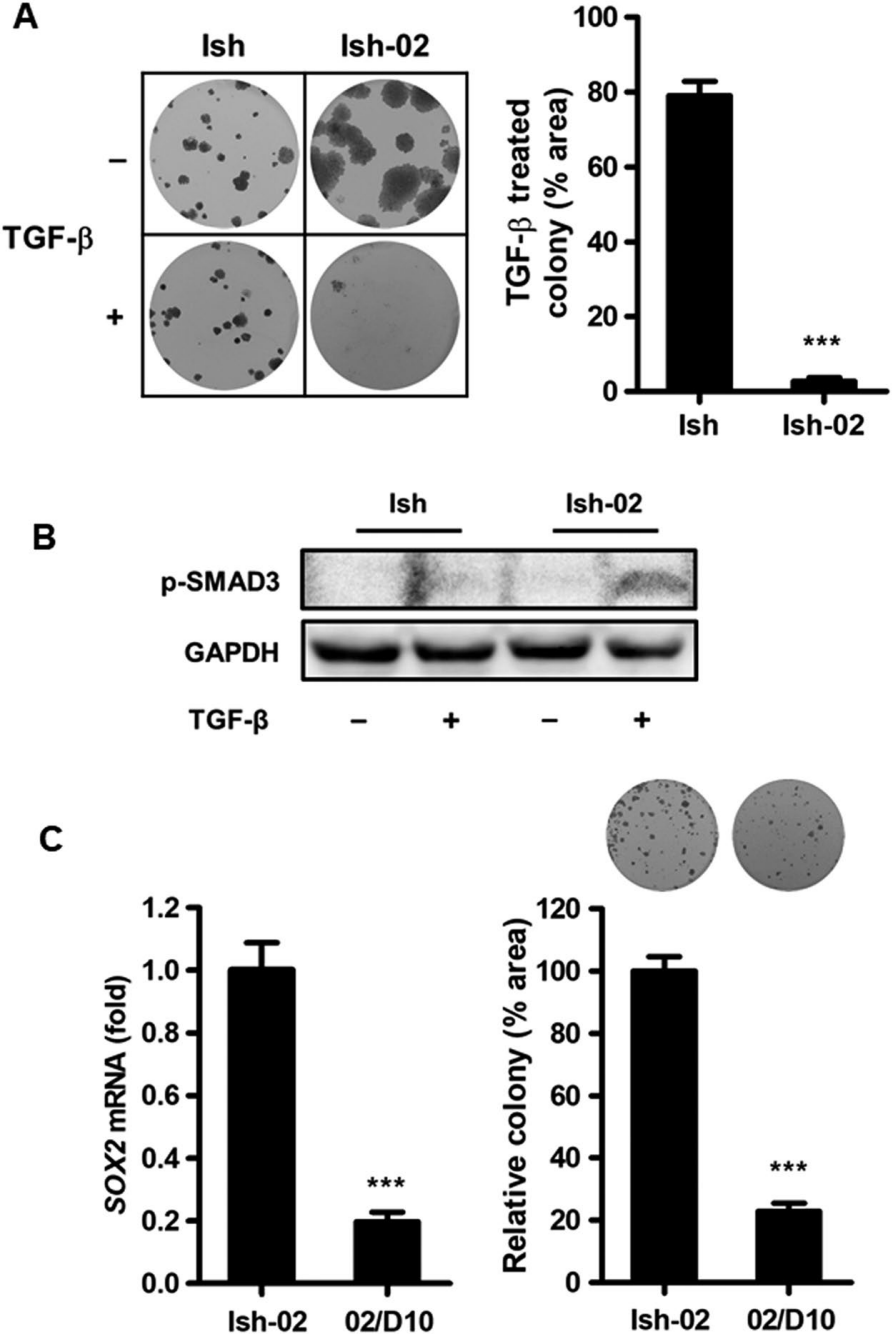
Effect of TGF-β stimulation on *SOX2* expression in endometrial carcinoma cells. (**A**) A clonogenic analysis to compare the growth of Ishikawa (Ish) and Ishikawa-02 (Ish-02) treated with or without TGF-β (1 ng/ml) for 14 days. Colonies were subjected to crystal violet staining (left) and quantified by ImageJ analysis (right). ***P < 0.001. (**B**) Immunoblotting analysis to access phosphorylated-SMAD3 (p-SMAD3) level in Ishikawa (Ish) and Ishikawa-02 (Ish-02) treated with or without TGF-β (1 ng/ml) for 4 hr. (**C**) Ishikawa-02 cells were first treated with TGF-β (1 ng/ml) for 10 days, and the treated cells were pooled together and named 02/D10. Q-PCR analysis (left) to measure SOX2 expression in Ishikawa-02 and 02/D10 cells. A clonogenic analysis (right) to compare the growth of Ishikawa-02 (Ish-02) to 02/D10 cells in the absence of TGF-β for 14 days. Colonies were subjected to crystal violet staining (upper) and quantified by ImageJ analysis (lower). ***P < 0.001.

TGF-β signaling inhibits proliferation of uterine epithelial cells^25,26^. To study the effect of TGF-β stimulation on SOX2-mediated growth in endometrial carcinoma cells, we treated Ishikawa and Ishikawa-02 (*SOX2*-high) cells with TGF-β and performed a clonogenic analysis (Fig. 4A). Compared with the Ishikawa cells, we observed that the growth of Ishikawa-02 cells was significantly inhibited by TGF-β treatment. Because decreased expression and frameshift mutations of TGFBR2 have been detected in endometrial carcinoma^29^, we further examined the mutation status of Ishikawa and Ishikawa-02 cells. The sequencing analysis showed that both Ishikawa and Ishikawa-02 harbor a heterogeneous frameshift mutation in the 10-bp poly(A) repeat of *TGFBR2* coding regions (Supplementary Figure S3A). To further identify the mechanism behind the distinct growth inhibitory effects of TGF-β on Ishikawa and Ishikawa-02 cells, we treated both cells with TGF-β, followed by immunoblotting for their phosphorylated-SMAD3 (p-SMAD) expression (Fig. 4B). We discovered that p-SMAD3 was induced by TGF-β in Ishikawa-02 but not in Ishikawa. A qPCR analysis further revealed that Ishikawa had a much lower *SMAD3* level compared to Ishikawa-02 and A549, which was adopted as a TGF-β-responsive positive control cell line (Supplementary Figure S3B). These data indicate that SMAD3 pathway is inhibited in Ishikawa but not in Ishikawa-02 cells.

TGF-β modulates normal endometrial growth and differentiation^30^, and antagonizes SOX2-mediated reprograming of fibroblasts into induced pluripotent stem cells (iPSC)^31^. To test whether TGF-β differentiation signaling affects *SOX2* expression in endometrial cancer, Ishikawa-02 (*SOX2*-high) cells were treated with TGF-β. A qPCR analysis revealed that TGF-β stimulation gradually downregulated *SOX2* level in Ishikawa-02 cells (Supplementary Figure S4A). After 10 days of TGF-β stimulation, the treated cells were pooled together and named 02/D10. We observed that 02/D10 had a much lower SOX2 expression than Ishikawa-02 cells (Fig. 4C, left). Ishikawa-02 and 02/D10 cells were further subjected to clonogenic assays in the absence of TGF-β stimulation. Compared with Ishikawa-02 cells, 02/D10 cells exhibited a worse colony-forming ability (Fig. 4C). These findings demonstrated that long-term TGF-β treatment suppresses *SOX2* expression and inhibits growth of endometrial carcinoma cells.

### SOX2 promotes migration in endometrial carcinoma cells

**Figure 5:**
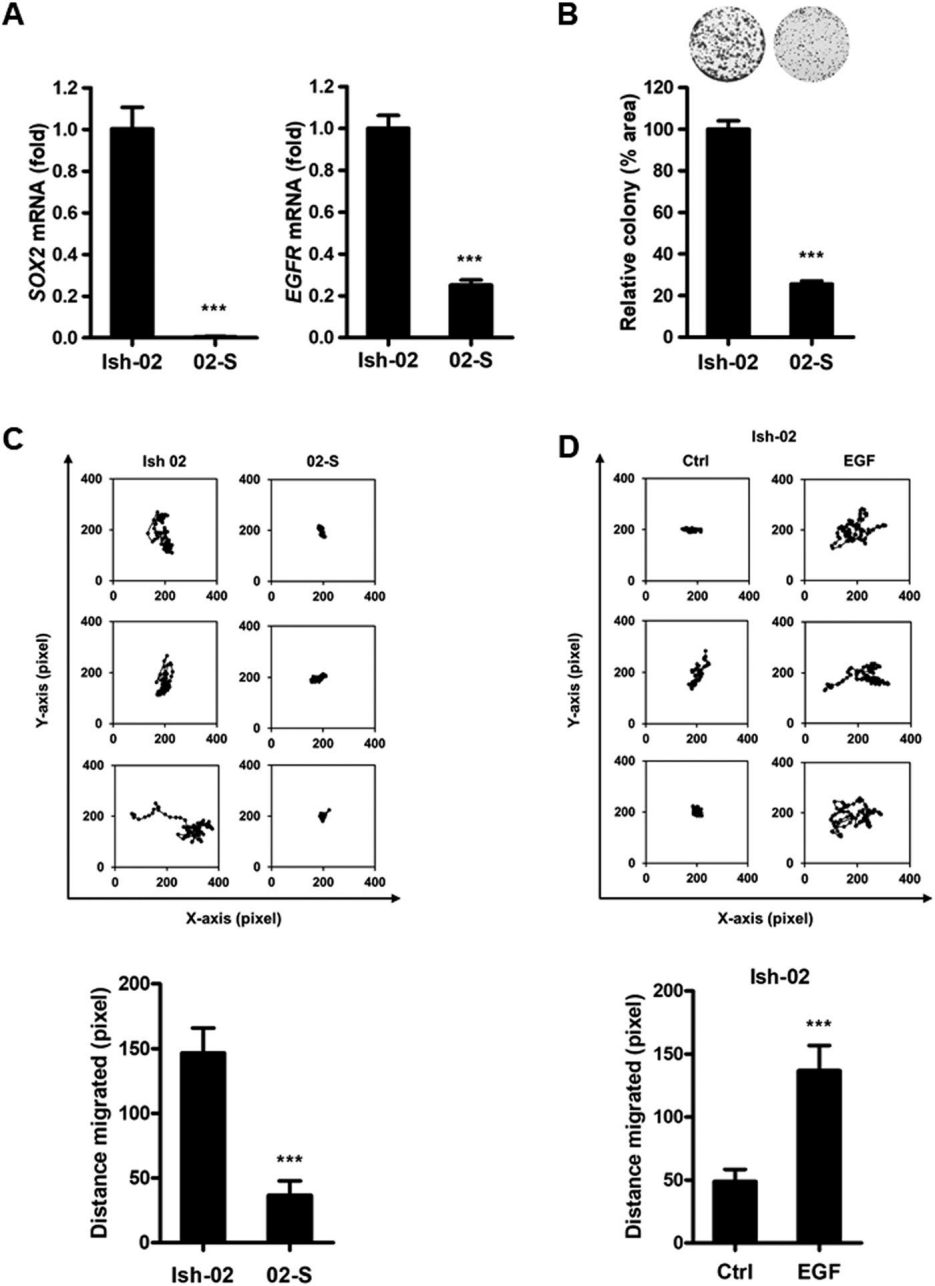
SOX2 promotes migration of endometrial carcinoma cells. (**A**) qPCR analysis to measure *SOX2* (left) and *EGFR* (right) expression in Ishikawa-02 (Ish-02) and Ishikawa-02-S (02-S) cells. Ishikawa-02-S is a stable clone selected from 02/D10, the pooled Ishikawa-02 cells previously treated with TGF-β (1 ng/ml) for 10 days. ***P < 0.001. (**B**) A clonogenic analysis to compare the growth of Ishikawa-02 (Ish-02) and Ishikawa-02-S (02-S) for 14 days. Colonies were subjected to crystal violet staining (upper) and quantified by ImageJ analysis (lower). ***P < 0.001. (**C**) Cell tracking analysis to monitor the trajectory (upper) and quantify migrated distance (lower) of Ishikawa-02 (Ish-02) and Ishikawa-02-S (02-S) cells under a normal serum condition. ***P < 0.001, n = 10 cells/group. (**D**) Cell tracking analysis to monitor the trajectory (upper) and quantify migrated distance (lower) of Ishikawa-02 cells treated with EGF (100 ng/ml) (EGF) for 24 hr under a serum starvation condition. ***P < 0.001, n = 10 cells/group.

To further study how the crosstalk between SOX2 and TGF-β stimulation affect the cell fate of endometrial carcinoma, we selected a stable clone from 02/D10 cells. Compared with the parental Ishikawa-02 cells, both *SOX2* and *EGFR* expression levels were downregulated in Ishikawa-02 stable clone (02-S) cells (Fig. 5A). Furthermore, the clonogenic analysis showed that 02-S cells grew slower than the parental Ishikawa-02 cells (Fig. 5B). The EOS-S(4+) reporter plasmid, which harbors *SOX2* regulatory region 2 (SRR2), was originally used to isolate endogenous SOX2-activated iPSC^32^. To enrich endogenous SOX2 signaling in 02/D10 (SOX2-low) cells, we used an EOS-S(4+) reporter plasmid to isolate SOX2-high clones. Through EOS-S(4+) reporter plasmid selection, five SOX2-high descendant clones (EOS4-1∼5) were isolated from 02/D10 cells (Supplementary Figure S4B). We observed a positive correlation between *SOX2* and *EGFR* expression in Ishikawa-02, 02/D10 and EOS4-1∼5 cells (Supplementary Figure S4B, S4C).

In addition to cell proliferation, EGFR signaling also regulates migration of progenitor cells^33^. To study the role of SOX2–EGFR signaling in the migration of endometrial carcinoma, we performed cell tracking analysis on Ishikawa-02 (*SOX2*-high) and 02-S (*SOX2*-low) cells. The results showed that 02-S cells exhibited a lower migratory ability than the parental Ishikawa-02 cells (Fig. 5C). The cell tracking analysis further revealed that EGF stimulation promoted migration of Ishikawa-02 cells (Fig. 5D). All these data indicate that SOX2–EGFR signaling promotes the migration of endometrial carcinoma cells.

### *SOX2* expression is associated with lymph node metastasis

**Figure 6:**
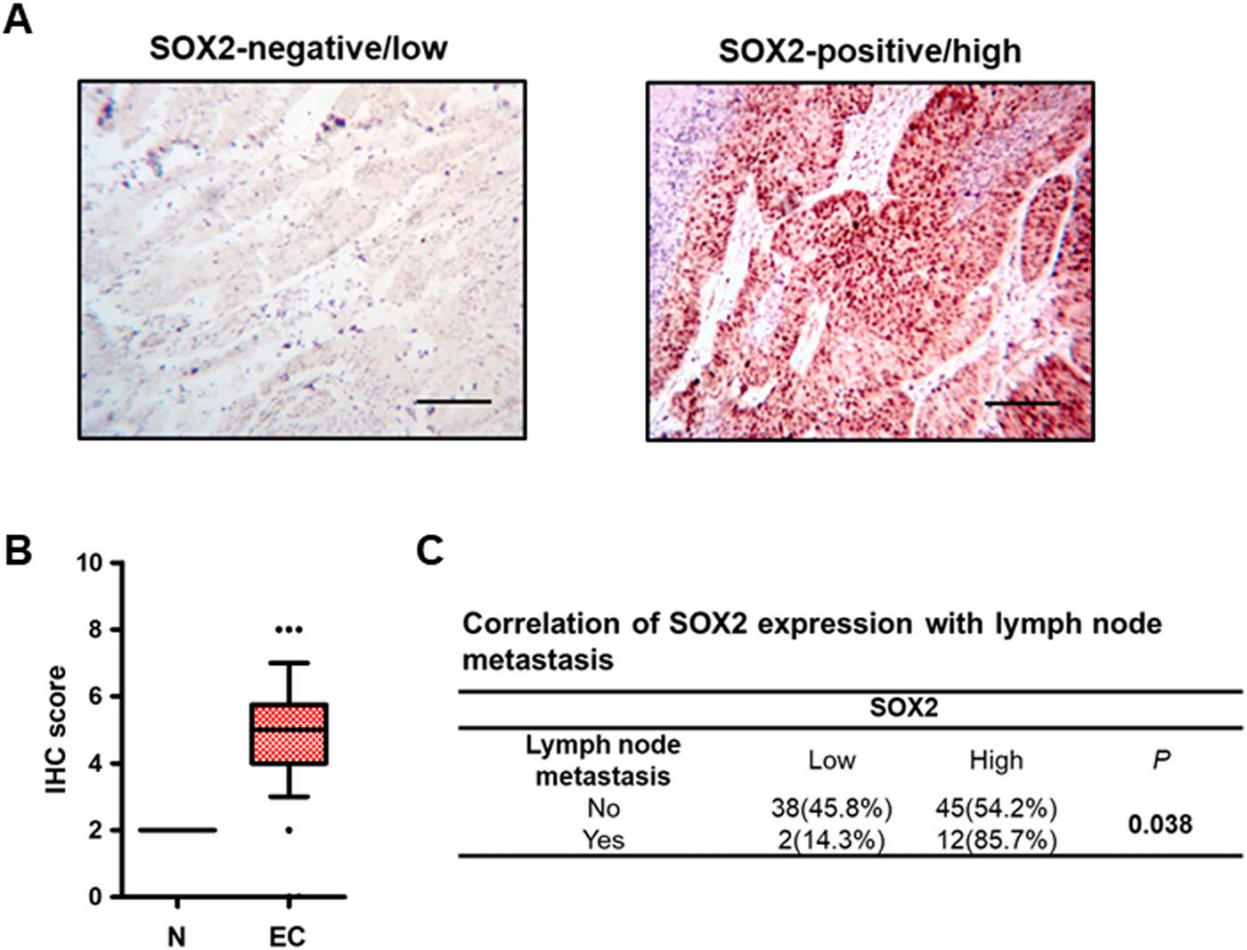
SOX2 expression correlates with lymph node metastasis of endometrial carcinoma. (**A**) Representative pictures of the immunohistochemistry analysis of SOX2 expression in endometrial carcinoma. A SOX2-negative/low case (left) and a SOX2-positive/high case (right) from an endometrial carcinoma tissue array. Scale bars = 100 μm. (**B**) Quantification analysis of SOX2 staining scores in 5 cases of normal tissue (N) and 97 cases of endometrial carcinoma tissues (EC). ***P < 0.001. (**C**) Correlation analysis of SOX2 expression with lymph node metastasis of endometrial carcinoma from the endometrial carcinoma tissue array.

The above data suggest the involvement of SOX2 in the dissemination of endometrial carcinoma. We performed an immunohistochemistry assay to detect SOX2 expression in normal endometrial tissues and endometrial carcinoma (Fig. 6A). Compared with normal endometrial tissues, we found that SOX2 was highly expressed in endometrial carcinoma (Fig. 6B). The correlation analysis further revealed that SOX2 expression correlated with lymph node metastasis (Fig. 6C). Moreover, we observed a positive association of *SOX2* with *EGFR* expression in stage IV metastatic endometrial carcinoma, but not in early stage cancer (Supplementary Figure S4D). These findings suggest that *SOX2* is involved in dissemination of endometrial carcinoma.

## Discussion

The stem cell factors SOX2, OCT4, and NANOG form positive feedback loops to maintain stemness in ESCs, and their involvement in tumor malignancies has been reported in several cancers^6,9–11^. However, their roles in endometrial carcinoma remain unclear. In this study, we observed that the expression of *SOX2*, but not *OCT4* and *NANOG*, correlates with poor histological differentiation of endometrial tumors, associates with lymph node metastasis, and predicts a poor survival in patients. In addition, we discovered that SOX2 induces EGFR in a positive feedback manner, contributing to cell cycle progression and migration of endometrial cancer cells. In contrast, TGF-β stimulation inhibits *SOX2* expression and thus suppresses the SOX2–EGFR-mediated effect in cells. Our data indicate that cytokine-mediated SOX2–EGFR signaling promotes malignancy in endometrial carcinoma.

The ESC-like signature has been correlated with poorly differentiated aggressive human tumors from breast, brain, and bladder^6^. In addition, MYC expression has been suggested to be essential to reactivate the ESC-like program and to be the main cause of tumor malignancy^7,8^. Thus, in this study, we checked the association of *SOX2*, *OCT4*, *NANOG*, and *MYC* expressions with histological differentiation of endometrial tumors, and observed that the expressions of *SOX2* and *MYC*, but not that of *OCT4* and *NANOG*, correlate with advanced tumor grades. Consistent with this finding, Pityński *et al*. reported that the expression of SOX2, but not that of OCT4, is related to tumor grading in endometrial carcinoma. We further identified the presence of genomic amplifications in *SOX2* and *MYC* in endometrial tumors, and we observed the association of *SOX2* and *MYC* amplifications with poor differentiation status. By analyzing the associations of *SOX2*, *OCT4*, *NANOG*, and *MYC* expressions with the survival outcomes in patients, we observed that *SOX2* and *MYC*, but not *OCT4* and *NANOG*, predict poor survival in patients. Moreover, *SOX2* expression is negatively associated with *MYC*. A multivariate analysis revealed that *SOX2* and *MYC*, but not *OCT4* and *NANOG*, are independent prognostic factors. These data suggest that *SOX2* and *MYC* are involved in different oncogenic signaling. Although *SOX2* expression is correlated with poor grade and prognosis, and is negatively associated with *ESR1*, which is higher expressed in endometrioid type than in serous type, we do not observe higher SOX2 expression in serous type than in endometrioid type (Supplementary Figure 2D). These findings suggest that *SOX2* can serve as an independent prognostic factor to predict endometrial carcinoma progression but may not be a suitable diagnostic marker for subtyping endometrial carcinoma.

SOX2 regulates self-renewal and differentiation in ESCs and adult progenitor cells^12–14^. Here, we observed that SOX2 contributes to cell cycle progression, thus increasing cell proliferation in endometrial carcinoma cells. During the menstrual cycle, EGFR signaling is highly activated to regulate the proliferation of endometrium epithelial cells^23^. We found that *SOX2* knockdown inhibited EGFR expression and attenuated cell proliferation in endometrial carcinoma cells. Moreover, *EGFR* silencing decreased *SOX2* level and suppressed cell growth. These data suggest that SOX2 and EGFR form a positive feedback loop, promoting cellular proliferation in endometrial carcinoma cells. In neuron progenitor cells, the SOX2–EGFR positive feedback loop is essential for self-renewal and proliferation^13^. The SOX2–EGFR positive feedback loop is also present in lung cancer cells to promote cell proliferation^17^. Our findings suggest that the SOX2–EGFR signaling is hijacked in endometrial carcinoma to promote cell cycle progression. In addition to cell proliferation, EGFR signaling contributes to progenitor cell migration and cancer dissemination^33–35^. We observed that SOX2 expression correlates with lymph node infiltration of endometrial tumors. We found that *SOX2*-high endometrial cancer cells migrate more than their *SOX2*-low counterparts. Moreover, EGF stimulation induced *SOX2* expression and promoted migration in endometrial cancer cells. We also observed a positive correlation between *SOX2* and *EGFR* expression in stage IV metastatic endometrial carcinoma, but not in early stage cancer. These data indicate the involvement of SOX2–EGFR signaling in cancer dissemination in endometrial carcinoma.

Overexpression of EGFR has been detected in endometrial carcinoma, correlating with advanced tumor grade, metastasis, and poor prognosis^36^. The therapeutic effect of gefitinib, an EGFR tyrosine kinase inhibitor, has been tested in GOG 229-C trial, which enrolled 29 women of whom 26 were evaluable under the treatment with 500 mg oral gefitinib daily. In this phase II trial, one patient, who did not harbor EGFR mutation, experienced a complete response. Although the predictive biomarker for gefitinib treatment in the endometrial carcinoma patient was not identified, patients having a stable disease over 6 months tented to have higher soluble EGFR^37^. Moreover, Leslie *et al*. reported that endometrial carcinoma cells harboring p53-null mutation are much more sensitive to the combinatorial treatment with gefitinib and paclitaxel than those harboring wild-type p53 or hotspot mutants^38,39^. Here, we observed that SOX2 level correlates with EGFR expression in late-stage endometrial tumors and cancer cells, in which SOX2 expression promotes EGFR, forming a SOX2–EGFR feedback. SOX2 expression has been known to enhance chemo-resistance ability in lung cancer^17,40^. In addition to serving as a prognostic marker, whether SOX2 can assist EGFR and p53 as a predictive marker in gefitinib single treatment or combinatorial treatment with paclitaxel in endometrial carcinoma requires further investigation.

In contrast to EGFR signaling, TGF-β inhibits growth of uterine epithelial cells^24^. Inhibition of TGF-β receptors (TGFBR) induces proliferation of uterine epithelial cells and mesenchymal stem cells^25,26^. Decreased expression and frameshift mutations of TGFBR2 have been detected in endometrial carcinoma^29^. Although a pro-metastatic role for TGF-β signaling in the pathogenesis of human endometrial carcinoma has been proposed mostly based on the cell line invasion model, Gao *et al*. deleted both Tgfbr1 and Pten in the mouse uterus to identify the potential role of TGF-β signaling in endometrial carcinoma^41^. They found that Pten/Tgfbr1 double knockout mice developed severe endometrial lesions that progressed more rapidly than those resulting from conditional deletion of Pten alone, suggesting that TGF-β signaling suppresses endometrial carcinoma progression^41^. From Cancer Cell Line Encyclopedia (Novartis/Broad, Nature 2012), inactivating frameshift mutations of TGFBR2 can be detected in endometrial cancer cell lines, such as COLO684, HEC59, HEC108, HEC151, HEC265, JHUEM1, JHUEM2, and RL95-2. Ishikawa cell line is an *ESR1*-positive well-differentiated human endometrial adenocarcinoma cell line composed of a heterogeneous mixture of endometrial cancer cells^42^, while Ishikawa-02 is an *ESR1*-negtaive cell line derived from Ishikawa. Here we report that Ishikawa, a SOX2-negtaive cell line, exhibits a lower proliferation rate than its SOX2-positive counterpart Ishikawa-02. Knockdown of SOX2 inhibited cell growth in Ishikawa-02 cells. We found that both Ishikawa and Ishikawa-02 harbor a heterogeneous inactivating frameshift mutation of TGFBR2. As TGF-β inhibits SOX2 signaling in iPSC^31^, we measured the effect of TGF-β stimulation on SOX2 expression in endometrial cancer cells. Since TGFBR2 is partially silencing mutated in Ishikawa and Ishikawa-02 cells, instead of adopting short-term TGF-β treatment for 24 hours used in the conventional invasion assay, we measured long-term TGF-β effect for 10 days on endometrial cancer cell growth and SOX2 expression. We observed that long-term TGF-β stimulation downregulated SOX2 and EGFR levels and inhibited cell growth in Ishikawa-02, while Ishikawa was not sensitive to TGF-β-mediated growth inhibition compared to Ishikawa-02. We found that Ishikawa contained much lower SMAD3 and p-SMAD3 levels than Ishikawa-02. Moreover, a wound healing assay showed that TGF-β cannot stimulate cellular migration in Ishikawa cells (Supplementary Figure S3C), indicating the loss of TGFBR2-SMAD3 signaling in Ishikawa. However, how SMAD3 levels are differentially regulated in Ishikawa and Ishikawa-02 requires further study. Together these data support the notion that TGF-β signaling plays a suppressive role in tumor progression and is inhibited in some of endometrial cancer cells.

In this study, we found that Ishikawa-02 exhibits cancer cell plasticity. The clonal cells isolated from long-term treatment of Ishikawa-02 cells with TGF-β contain low levels of both *SOX2* and *EGFR*. By enriching endogenous SOX2 expression via the EOS-S(4+) reporter plasmid selection from SOX2-low cells, we obtained SOX2-high descendant clones and observed that *SOX2* expression correlates with *EGFR* level in these cells. All these data support the existence of the SOX2-EGFR positive feedback in some, if not all, of endometrial cancer cells. These findings also suggest that *SOX2* is regulated by EGFR and TGF-β signaling, and that deregulated *SOX2* expression and cytokine signaling may promote endometrial tumor malignancy.

Taken together, we found that *SOX2* is highly expressed in endometrial carcinoma, in which SOX2 crosstalks with cytokine signaling to promote cell proliferation and migration, thereby predicting poor survival. These findings provide new insights into the role of *SOX2*, a stem cell factor, in oncogenesis, with the potential to serve as a biomarker in endometrial carcinoma.

## Materials and Methods

### Cell lines

Ishikawa cells (ECACC No. 99040201) and Ishikawa (Heraklio) 02 ER- cells (ECACC No. 98032302) were obtained from the European Collection of Authenticated Cell Cultures as described previously^43^. Ishikawa cells harbor estrogen receptors (ER+) and were originally established from an endometrial adenocarcinoma from a 39 year old woman with the ability to induce well differentiated adenocarcinoma in the xenograft nude mice model^44^. Ishikawa (Heraklio) 02 ER-, shortened as Ishikawa-02, is an estrogen receptor-negative (ER-) descendant from Ishikawa cells (https://web.expasy.org/cellosaurus/CVCL_2529). All cells were cultured in DMEM supplemented with 10% fetal bovine serum (FBS).

### Plasmid Construction

SOX2 expressing vector and EGFR promoter reporter were prepared as described previously^17^. pLKO.1-shSOX2#1 (TRCN0000003253), pLKO.1-shSOX2#2 (TRCN0000010772), pLKO.1-shEGFR#1 (TRCN0000039634), and pLKO.1-shEGFR#2 (TRCN0000121067) clones were obtained from the National RNAi Core Facility, Academia Sinica (Taipei, Taiwan). pLKO.1-Scrambled control shRNA were obtained from Addgene.. Lentiviral production and infection were performed using previously described methods^45^.

### Reagents

Recombinant human EGF and TGF-β1 (TGF-β) were purchased from Sino Biological Inc. (Beijing, China).

### Quantitative real-time polymerase chain reaction (qPCR)

qPCR was performed with specific primers and taqman probes or probes from the Universal Probe Library (Roche Applied Science) in the StepOne^TM^ Real-Time PCR system (Applied Biosystems Inc., Foster City, CA)^46^. Primer sequences designed to detect specific genes and probes are listed in Supplementary Table S2. 18 S rRNA was used as a reference transcript.

### Tissue samples and public Domain Data Analysis

The specimens used in immunohistochemistry analysis were obtained from a high-density tissue array (EMC1021, US BIOMAX). The public gene expression profiling data sets used in this study were analyzed as described previously^40,46^. Briefly, the expression of specific gene (*SOX2*, *OCT4*, *NANOG*, and *MYC*) and survival information from TCGA_UCEC^27^ were used for the Kaplan–Meier overall survival analysis with the medium levels of these genes as cut-off points. The gene expression and tumor grading information from TCGA_UCEC and GSE17025^47^ datasets were used for correlation analysis. Public Domain Data Analysis. The sources of these gene expression profiling datasets are listed in Supplementary Table S3.

### Clonogenic Assay

Cells were seeded in 6-well plates (1000 cells per well) or 24-well (100 cells per well). Cells were incubated for 2 weeks at 37 °C with 5% CO_2_. The medium was aspired and the surviving colonies were fixed and stained with crystal violet (Fisher Scientific). Colonies were counted and measured by ImageJ software.

### TrypanBlue Cell Exclusion Assay

Cells (10^5^ cells/well) were seeded in 24-well plates and incubated in triplicate tests. In indicated times, cells were trypsinized and then counted using Trypan Blue (Gibco) staining under manufacture instructions in low magnification under microscopy.

### Cell Tracking Analysis

Cells (2 × 10^5^ cells per well) were seeded in 6-well plates and incubated for 24 h. LS620 Microscope (Lumascope) was used to record single cell position every 10 min for 24 h. Single cell migration distance and trajectory were analyzed by ImageJ.

### Immunoblotting

Cells were harvested in RIPA lysis buffer supplemented with a protease inhibitor cocktail, and immunoblotting was performed with SOX2 antibody (1: 1000 dilution, GTX101506, GeneTex).

### Immunohistochemistry (IHC) Staining

IHC was conducted as previously described^17^. The primary SOX2 antibody used in the IHC was SOX2 antibody (1:300 dilution, PM056, MBL). The immunoreactivity pattern and histologic appearance of all clinical specimens and tissue microarray slides were examined and scored by the pathologist. The final score was determined by the sum of the intensity scores (0 = no staining, 1 = week, 2 = moderate, 3 = strong staining) and the positivity scores of stained cells (0 = 0%, 1 < 1%, 2 = 1–10%, 3 = 11–33%, 4 = 34–66%, 5 = 67–100%). The total score of 0–4 is divided into low performance, and 5–8 is divided into high performance.

### Statistical Analysis

The association between specific gene expression and clinicopathological parameters was evaluated using Pearson’s chi-square test. Survival curves were compared using the log-rank test. The prognostic significance of the association of specific expression with overall survival was assessed using the multivariate Cox’s proportional hazards analysis. The relative expression of specific genes among different tumor grades was compared using analysis of variance. All statistical analyses were performed using SPSS software, version 16 (SPSS, Inc., Chicago, IL, USA). A value of *P* < 0.05 was considered statistically significant.

## Electronic supplementary material

Supplementary Information
