# Supplementary material for: Crosstalk between SOX2 and cytokine signaling in endometrial carcinoma
Source: Sci Rep. 2018 Dec 3;8:17550. doi: 10.1038/s41598-018-35592-0 (PMC6277382; doi:10.1038/s41598-018-35592-0)
Supplement: Supplementary file 1 — Supplementary Information [file 41598_2018_35592_MOESM1_ESM.pdf]

Supplementary information

Crosstalk between SOX2 and cytokine signaling in endometrial carcinoma

Chang-Jung Lee, Pi-Lin Sung, Ming-Han Kuo, Min-Hwa Tsai, Cheng-Kuan Wang, Shien-Tung Pan, Yi-Jen Chen, Peng-Hui Wang, Kuo-Chang Wen, and Yu-Ting Chou

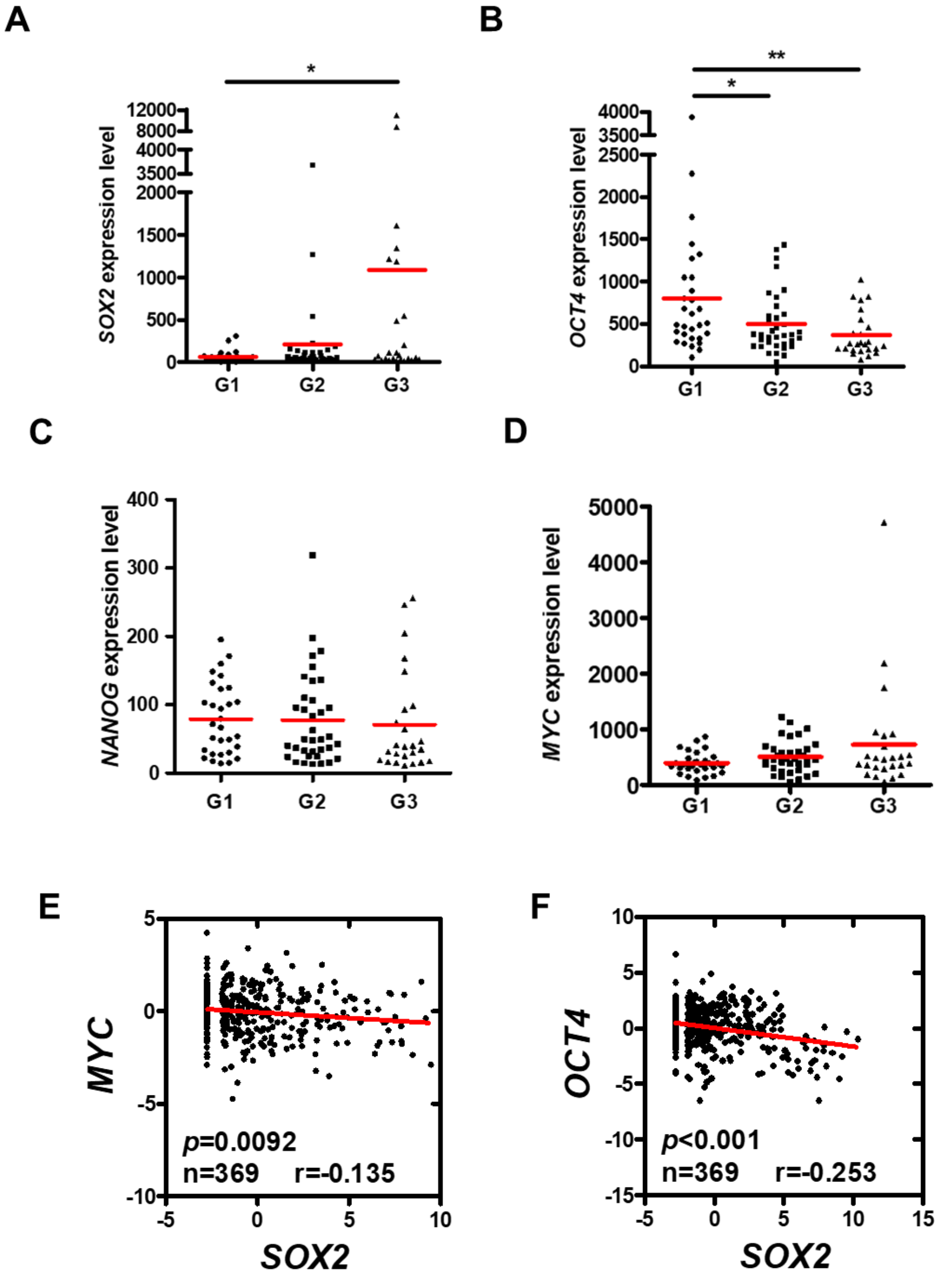

Figure S1. ***SOX2*** **and** ***OCT4*** **expression correlate with high and low histological grades, respectively.**

A-D. Gene expression analysis of (A) *SOX2*, (B) *OCT4*, (C) *NANOG*, and (D) *MYC* expression in different histological grades of endometrial carcinoma from GSE17025 cohort. The significance was examined by Tukey's Multiple Comparison Test followed by one way ANOVA. *, $P<0.05$; **, $P<0.01$. ***E***. Correlation analysis of *SOX2* and *MYC* expression in endometrial carcinoma from TCGA_UCEC cohort. ***F***. Correlation analysis of *SOX2* and *OCT4* expression in endometrial carcinoma from TCGA_UCEC cohort.

Table S1. Univariate and multivariate analysis of *SOX2*, *OCT4*, *NANOG*, and *MYC* expression on overall survival of endometrial carcinoma in TCGA_UCEC cohort (n=369).

Variable		Univariate analysis		Multivariate analysis	
		Hazard ratio (± 95% CI)	*P*	Hazard ratio (± 95% CI)	*P*
SOX2	< -0.85884	1	0.003**	1	0.002**
	≥ -0.85884	2.831(1.426-5.618)		2.928(1.473-5.861)	
OCT4	< 0.150802	1	0.042*	1	0.108
	≥ 0.150802	0.521(0.278-0.976)		0.590(0.310-1.123)	
NANOG	< -0.01567	1	0.638	1	0.51
	≥ -0.01567	1.156(0.632-2.114)		1.232(0.662-2.294)	
MYC	< -0.02286	1	0.043*	1	0.032*
	≥ -0.02286	1.894(1.020-3.516)		1.999(1.060-3.769)	

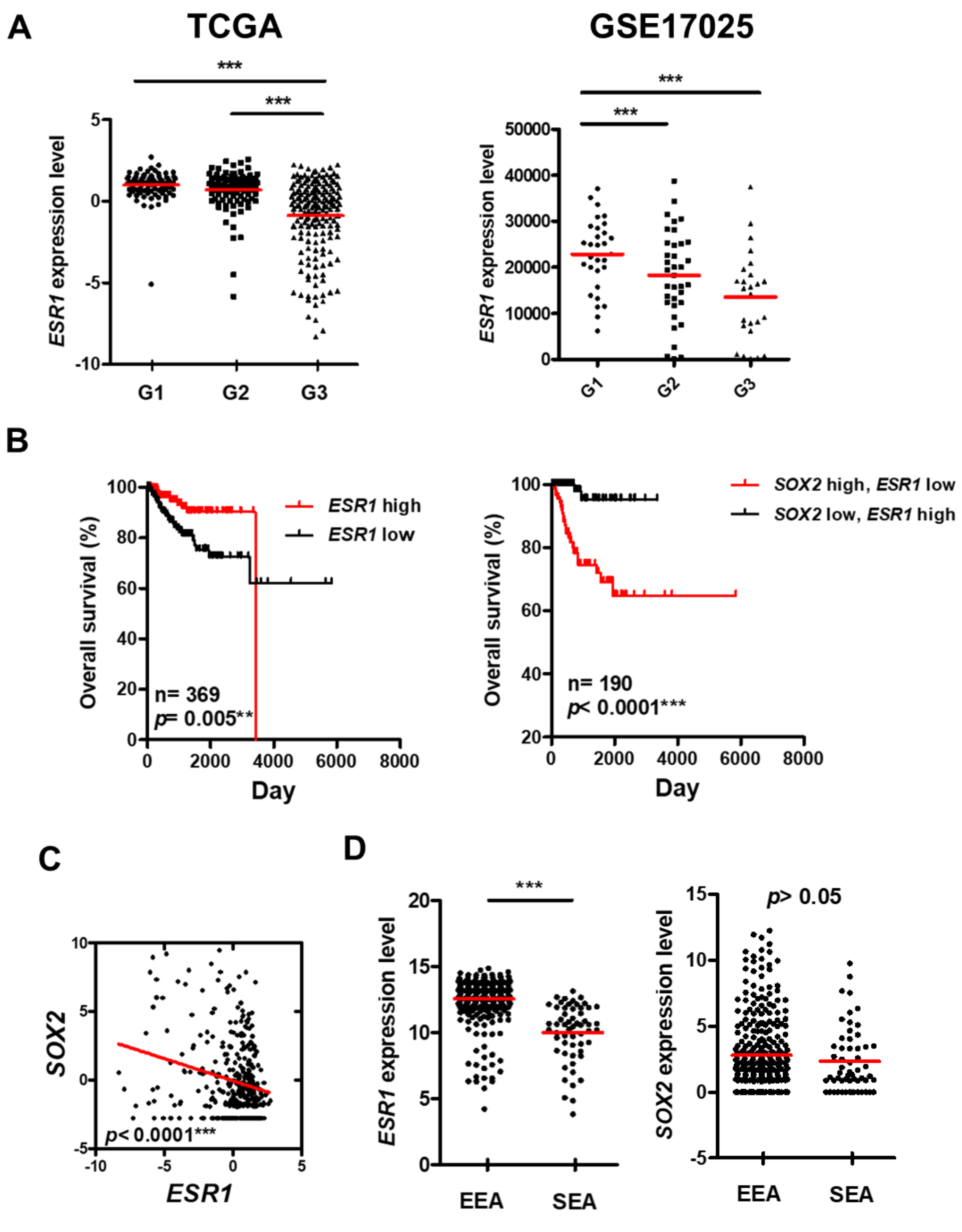

Figure S2. ***ESR1*** **and** ***SOX2*** **as prognostic factors in endometrial carcinoma.**

A. Gene expression analysis of *ESR1* in different histological grades of endometrial carcinoma from TCGA_UCEC (left) and GSE17025 (right) cohort. The significance was examined by Tukey's Multiple Comparison Test followed by one way ANOVA. ***, P<0.001. ***B.*** Kaplan–Meier analysis (left) to assess the correlation of *ESR1* expression with the overall survival of patients with endometrial carcinoma from TCGA_UCEC cohort. The significance was examined by log-rank test. Kaplan–Meier

analysis (right) to assess the correlation of *SOX2*-high/*ESR1*-low and *SOX2*-low/*ESR1*-high signatures with the overall survival of patients with endometrial carcinoma from TCGA_UCEC cohort. The significance was examined by log-rank test. ***C.*** Correlation analysis of *ESR1* with *SOX2* expression in endometrial carcinoma from TCGA_UCEC cohort. ***D***. Gene expression analysis of *ESR1* (left) and *SOX2* (right) in endometrioid endometrial adenocarcinoma (EEA) and serous endometrial adenocarcinoma (SEA) from TCGA_UCEC cohort. ***, P<0.001.

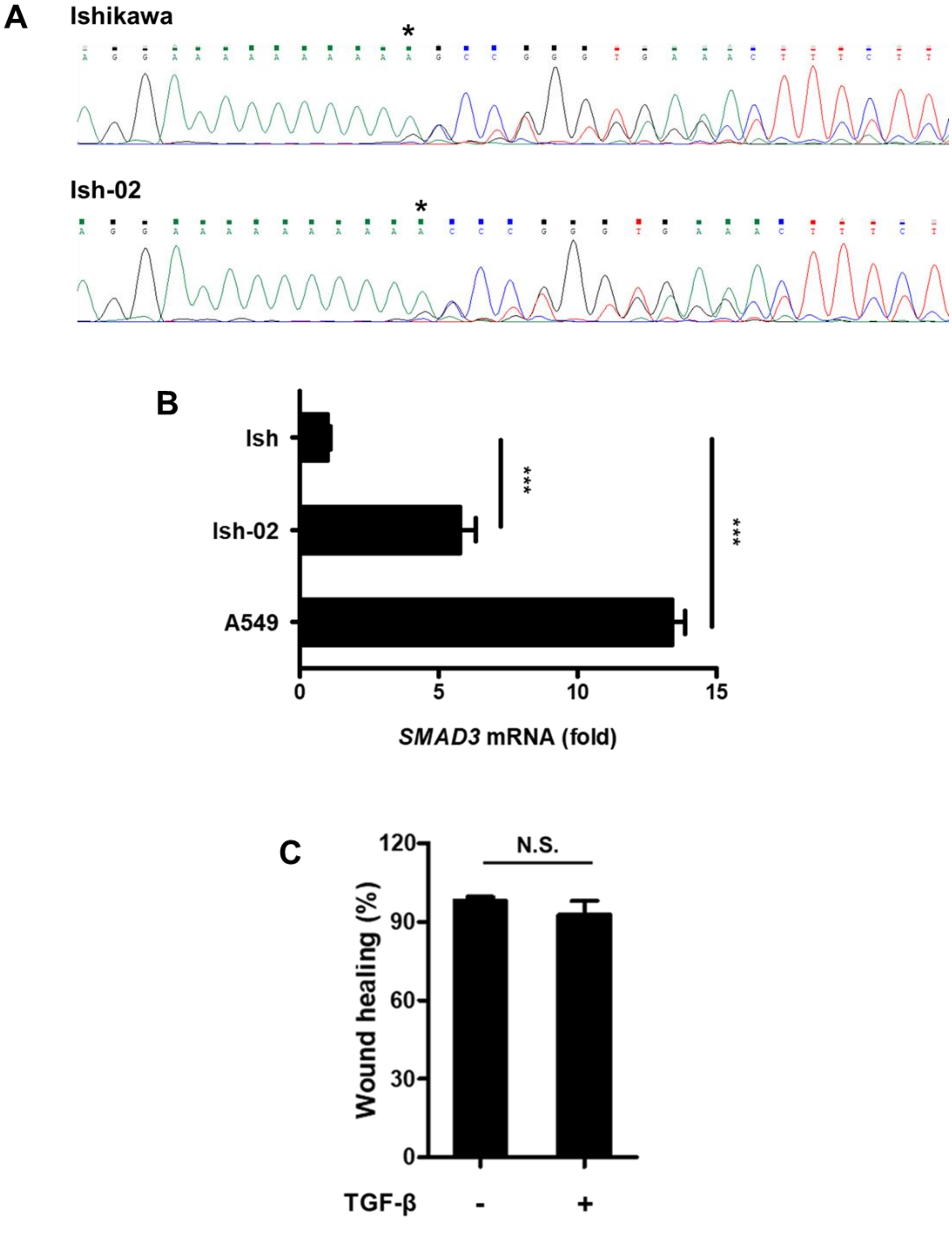

Figure S3. Loss of TGFBR2-SMAD3 signaling in Ishikawa cells.

A. DNA sequencing analysis of TGFBR2 in Ishikawa and Ishikawa-02 (Ish-02) cells. An asterisk indicates the position of a frameshift mutation in the 10-bp poly(A) repeat of TGFBR2 coding regions in both Ishikawa and Ishikawa-02. ***B.*** qPCR analysis to access SMAD3 level in Ishikawa (Ish), Ishikawa-02 (Ish-02) and A549 cells. ***, $P<0.001$. ***C.*** Wound healing analysis of Ishikawa cells treated with or without TGF-β (1 ng/ml) for 24 hr.

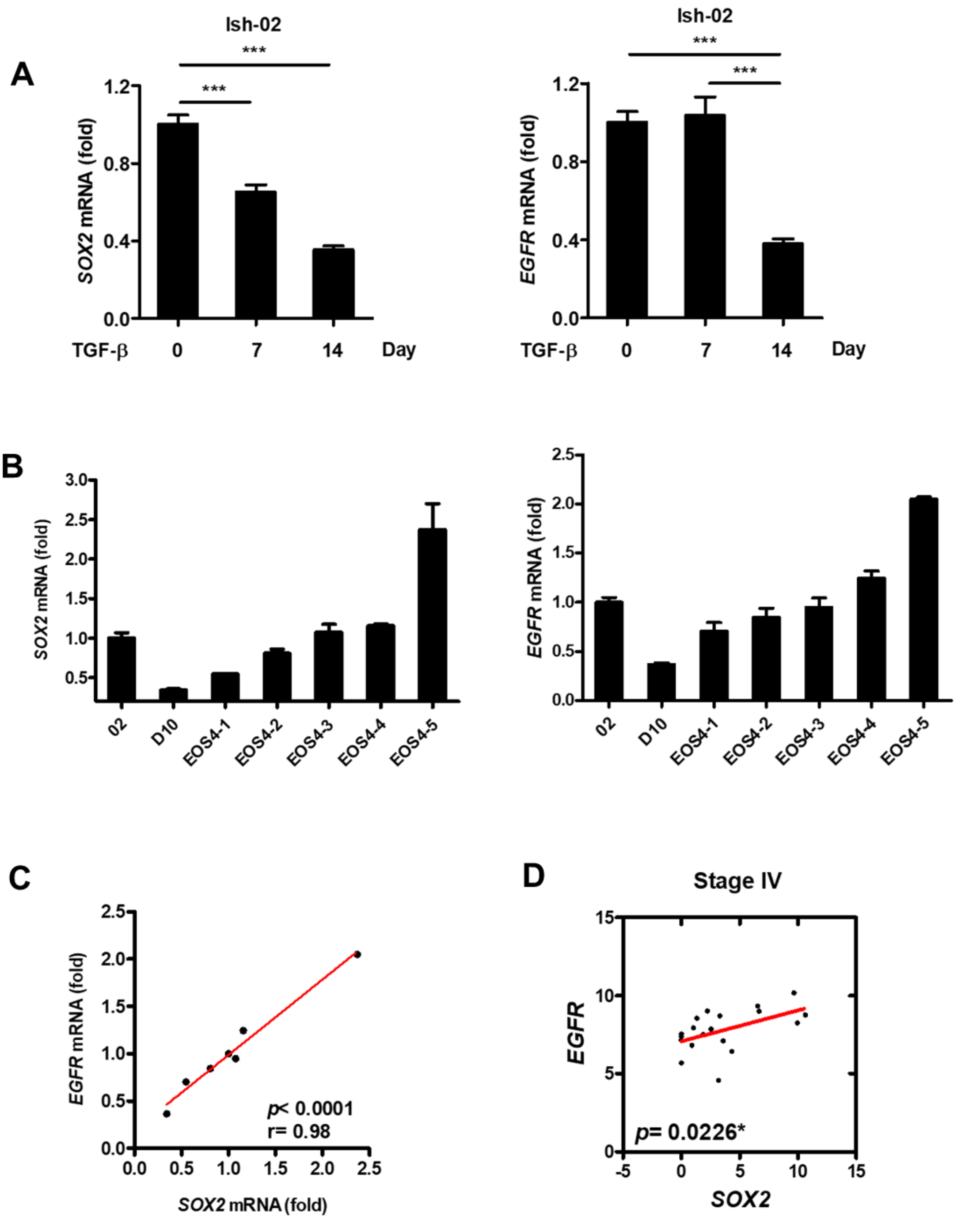

Figure S4. SOX2 correlates with EGFR expression in endometrial carcinoma.

A. qPCR analysis to access SOX2 (left) and EGFR (right) levels in Ishikawa-02 cells treated with TGF-β (1 ng/ml) for the indicated time periods. ***, P<0.001. ***B.*** qPCR analysis to access SOX2 (left) and EGFR (right) levels in Ishikawa-02, 02/D10, and EOS4-1~5 cells. EOS4-1~5, five SOX2-high clones, were identified by 02/D10 (SOX2-low) cells transduced with EOS-S(4+) lentiviral vectors and followed by puromycin selection. ***C.*** A scatter plot generated from Ishikawa-02, 02/D10, and

EOS4-1~5 cells displaying a positive correlation between SOX2 and EGFR levels. ***D.*** Correlation analysis of SOX2 with EGFR expression in Stage IV metastatic endometrial carcinoma from TCGA_UCEC cohort.

Table S2. Primer and probe list.

Primer/probe name	Sequences(5'→3')
18S forward primer	TGG CTC ATT AAA TCA GTT ATG
18S reverse primer	CGG CAT GTA TTA GCT CTA
18S probe	HEX-CGC TCG CTC CTC TCC TAC TTG-BHQ1
SOX2 forward primer	GCA GTA CAA CTC CAT GAC
SOX2 reverse primer	GAG GAA GAG GTA ACC ACA
SOX2 probe	FAM-CGC AGA CCT ACA TGA ACG GC-BHQ1
EGFR forward primer	TCC CCG TAA TTA TGT GGT GAC
EGFR reverse primer	AGG CCC TTC GCA CTT CTT AC
EGFR probe	UPL#69
ESR1 forward primer	TTA CTG ACC AAC CTG GCA GA
ESR1 reverse primer	ATC ATG GAG GGT CAA ATC CA
ESR1 probe	UPL#24
SMAD3 forward primer	CAT GGC ATG CAC GTA TGT AA
SMAD3 reverse primer	GAG GCC TGA GGC TAA GAA TG
SMAD3 probe	UPL#60

Table S3. Gene expression profiling data from the public domain used in this study

Cohort	Case #	Sources	References
TCGA_UCEC	596	TCGA	(1)
GSE17025	103	GEO	(2)

(1) https://tcga-data.nci.nih.gov/docs/publications/ucec_2013/

(2) https://www.ncbi.nlm.nih.gov/geo/query/acc.cgi?acc=GSE17025
